# Exploring socio-economic dimensions in HIV research: a comprehensive bibliometric analysis (1992–2024)

**DOI:** 10.1080/16549716.2025.2474787

**Published:** 2025-03-12

**Authors:** Lyudmila Yermukhanova, Marat Kuzembayev, Akkumis Salkhanova, Nazerke Narymbayeva, Aigul Tazhiyeva, Dinara Nurgalievna Makhanbetkulova, Alireza Afshar

**Affiliations:** a Department of Medicine, West-Kazakhstan Marat Ospanov Medical University, Aktobe, Kazakhstan; b Department of Nutrition, Kazakh Academy of Nutrition, Almaty, Kazakhstan; c Department of Medicine, Kazakhstan Medical University “KSPH”, Almaty, Kazakhstan; d Department of Medicine, Kazakh National Medical University Named After S.D. Asfendiyarov, Almaty, Kazakhstan; e Department of Nursing, Kazakh National Medical University, Almaty, Kazakhstan

**Keywords:** HIV infection, social determinants of health, global health, acquired immunodeficiency syndrome, health status

## Abstract

The socio-economic burden of HIV infection remains a critical global health concern. This study was conducted to perform a comprehensive bibliometric analysis of the socio-economic burden of HIV infection, highlighting research trends, collaboration networks, and the evolving focus on social determinants of health over the past 32 years. A systematic search was conducted in Scopus and Web of Science Core Collection databases, covering publications from 1992 to 2024. The analysis was performed using RStudio and Biblioshiny, focusing on 1,054 studies from 422 publications. This study revealed a steady annual growth rate of 16.72% in publications on the socio-economic burden of HIV from 1992 to 2024, with the USA and Canada leading in contributions. The University of Toronto emerged as the top institution, while ‘social determinants of health’ and ‘HIV infections’ were identified as pivotal research themes. Collaboration networks were predominantly among high-income countries, with limited engagement from high-burden regions like sub-Saharan Africa. Key journals, such as AIDS and Behavior, were identified as central to advancing the field. Thematic analysis highlighted a shift from biomedical to socio-economic factors, emphasizing the need for equitable global collaboration and research addressing disparities in HIV management. This comprehensive analysis provides valuable insights into the evolving landscape of HIV socio-economic burden research, emphasizing the need for increased collaboration with high-burden regions and a continued focus on addressing social determinants of health in HIV management.

## Paper Context

- **Main findings:** This study reveals a transformative shift in HIV research, from a predominantly biomedical focus to a deeper exploration of socio-economic dimensions, uncovering critical gaps in collaboration with regions bearing the highest HIV burden, such as sub-Saharan Africa.
- **Added knowledge:** By conducting the first comprehensive bibliometric analysis of HIV’s socio-economic burden over three decades, this paper illuminates emerging research trends, highlights underexplored themes, and identifies the need for equitable global collaboration to address the epidemic’s multifaceted impact.
- **Global health impact for policy and action:** The findings underscore the urgent necessity for policies that address socio-economic disparities in HIV management and call for strengthening research partnerships with high-burden regions to foster impactful, inclusive global health strategies.

## Background

The Human Immunodeficiency Virus (HIV) is a retrovirus that primarily targets the immune system, specifically CD4 cells or T-helper cells. By integrating its RNA into the host cell’s DNA, HIV disrupts the immune system, making individuals more vulnerable to infections and certain cancers [1]. Over time, HIV can lead to Acquired Immunodeficiency Syndrome (AIDS), a more advanced stage of the virus marked by severe immune system damage [2]. Globally, HIV continues to be a significant public health issue, affecting approximately 38 million people as of 2020, with Sub-Saharan Africa bearing a disproportionate burden, accounting for nearly 70% of cases [3,4]. The prevalence of HIV varies widely, with some regions showing infection rates of over 20% in the adult population, particularly in Southern Africa [3,4].

HIV infection imposes a considerable disease burden globally. In 2020, 1.5 million people were newly infected, and 680,000 people died from HIV-related causes, reflecting a persistent challenge despite advances in antiretroviral therapy (ART) [5]. Mortality rates have declined with ART, yet life expectancy for people living with HIV remains shorter than for the general population. Without treatment, life expectancy post-HIV infection can drop to around 8–10 years [3,4], but with ART, this can increase significantly, though it is still often several years shorter compared to HIV-negative individuals [1,6].

The socio-economic burden of HIV is profound, impacting not only individuals but also entire communities and health systems. For individuals, the cost of ongoing treatment, potential loss of income, and the stigma associated with HIV can lead to poverty and social exclusion [7]. At the national level, countries with high HIV prevalence experience decreased workforce productivity, increased healthcare expenditures, and a slowdown in economic growth. Globally, the HIV epidemic has been estimated to reduce the economic growth rate in heavily affected countries by as much as 1% annually [8,9].

A thorough understanding of the global and local landscape of HIV through comprehensive data collection is essential for improving disease management and intervention strategies. Detailed epidemiological data enable policymakers and health-care providers to allocate resources efficiently, identify populations most at risk, and tailor intervention programs to local needs. Moreover, global surveillance of HIV trends can inform international collaborations and funding initiatives aimed at combating the disease more effectively [3,4,10].

Bibliometric analysis offers a powerful tool for tracking the progress of HIV research and identifying emerging trends. By analyzing patterns in scientific publications, co-authorship networks, and keyword frequency, bibliometrics provides a snapshot of the research landscape over time [11,12]. This approach allows researchers to map collaboration networks, track the evolution of research themes, and assess the impact of key studies within the field [11,12]. In the context of HIV, such analysis can help highlight shifts in focus, such as the growing attention to social determinants of health, and identify gaps where further research is needed [1]. The aim of the study was to provide a comprehensive analysis of the socio-economic burden of HIV infection, using bibliometric methods to explore research trends, collaboration networks, and emerging themes over time.

## Methods

### Study design and data collection

For a comprehensive investigation into the socio-economic burden of HIV infection, we undertook a systematic search, focusing on literature drawn from two major databases: Scopus and the Web of Science Core Collection (WOS-CC). Our methodology aimed to cover a broad spectrum of studies related to this field. Data extraction was carried out in October 2024. The search strategy was based on key terms such as ‘HIV’, ‘Human Immunodeficiency Virus’ and ‘Social Determinants of Health’ applied within either the title or abstract (see Table S1). The timeframe for the search was extended from 1992 to 2024, without imposing any specific time limitations, in line with the indexing protocols of the Scopus and WOS databases. Once the data from both databases were retrieved, they were combined, carefully cross-checked, and duplicates were removed to ensure the accuracy of our dataset.

**Figure 1.**
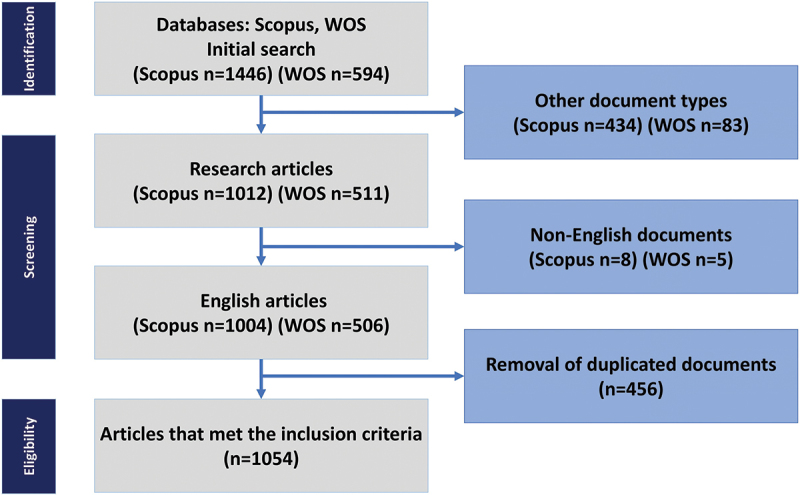
A schematic illustration of the screening process conducted in accordance with the PRISMA guidelines.

For the scope of this review, we focused exclusively on original research articles published in English. Publications that fell outside this domain, such as commentaries, editorials, or unrelated studies, were excluded from further analysis. Figure 1 outlines the full selection process.

- Inclusion criteria for search strategy:

- Articles published in English.
- Only original research articles.
- No timeline limitation.
- Studies specifically addressing the socio-economic burden of HIV infection, including themes like social determinants of health and HIV-related disparities.
- Articles indexed in the Scopus and Web of Science Core Collection databases.
- Studies with keywords such as ‘HIV,’ ‘Human Immunodeficiency Virus,’ and ‘Social Determinants of Health’ in the title or abstract.

- Exclusion criteria for search strategy:

- Articles published in languages other than English.
- Non-research articles, including editorials, commentaries, conference abstracts, letters, and unrelated studies.
- Studies not focused on the socio-economic dimensions of HIV or those unrelated to the targeted research themes.
- Duplicate records across Scopus and Web of Science databases.
- Articles with missing essential bibliometric information required for analysis (e.g. no author, no keywords, or no abstract).
- Publications outside the indexing scope of Scopus and Web of Science Core Collection.

### Analysis with R studio and Biblioshiny

The bibliometric analysis was executed using Rstudio (version 4.9.0), a versatile open-source software, paired with the bibliometric R package (R codes utilized for this study are shown in Table S2). The analysis was conducted on 7 October 2024, utilizing the Biblioshiny interface. Biblioshiny enhances the user experience by providing a more intuitive platform for conducting bibliometric research, facilitating the exploration of publication trends, collaboration networks, and keyword associations [11]. We employed co-authorship networks to uncover patterns of collaboration between researchers. Additionally, a keyword co-occurrence analysis was conducted to identify clusters of frequently occurring terms, revealing significant research themes. Citation analysis was also performed to determine the influence and scholarly impact of key articles within the field.

### Mapping key institutions, authors, and international collaborations

To further understand the relationships within the research landscape, we developed visual representations to map the most prolific institutions and authors involved in this field. These visualizations offered insights into the degree of collaboration between these entities. Countries were also ranked based on their research output, measured as the percentage of total publications. We additionally assessed the collaborations among the top 10 most productive nations, constructing a global map to depict the frequency and intensity of international collaborations.

### Keyword analysis and trend identification

We conducted a temporal analysis to monitor the evolution of keyword usage over time, allowing us to track how certain terms gained prominence throughout the years. A TreeMap was generated to visually represent the top 10 most frequently occurring keywords within the dataset. In addition, a detailed thematic analysis was performed, providing deeper insights into the main trends and prevailing research themes in the selected body of the literature.

## Results

### Overview of the studies

This research provides a comprehensive examination of the critical needs for socio-economic burden of HIV infection, assessing relevant publications from the period of 1992 to 2024. A total of 1054 studies were meticulously analyzed, sourced from 422 distinct publications. These contributions came from 6289 different authors, with an average citation rate of 14.26 citations per document over the last decade. The most influential findings regarding socio-economic burden of HIV infection were drawn from the most frequently cited papers of the last 10 years (Table 1). Further more, the annual growth rate for this field was calculated at 16.72% up to 2024, showing a steady increase in publications throughout the study period. The substantial academic output is also highlighted by the presence of 19,454 references and 2208 unique author keywords. Collaborative efforts among authors were notable but limited, accounting for only 12.62% of the studies.

**Table 1.** The top 10 most cited documents on socio-economic burden of HIV infection from 1992 to 2024.

Rank	Study ID [References]	Title of the Document	Journal Name	Total Citations	DOI
1	O’Neill J, [2],	Applying an equity lens to interventions: using PROGRESS ensures consideration of socially stratifying factors to illuminate inequities in health	Journal of Clinical Epidemiology	716	10. 1016/j.jclinepi.2013.08.005
2	Pillay-Van Wyk V, [1],	Mortality trends and differentials in South Africa from 1997 to 2012: second National Burden of Disease Study	The Lancet Global Health	265	10. 1016/S2214-109X(16)-9
3	Aidala Aa, [7],	Housing status, medical care, and health outcomes among people living with HIV/AIDS: a systematic review	American Journal of Public Health	264	10. 2105/AJPH.2015.302905
4	Shiau S, [13],	The burden of COVID-19 in people living with HIV: a syndemic perspective	AIDS and Behavior	251	10. 1007/s10461-020 -02,871-9
5	Reisner Sl, [14],	Integrated and gender-affirming transgender clinical care and research	Journal of Acquired Immune Deficiency Syndromes	218	10. 1097/QAI.0000000000001088
6	Stockman Jk, [15],	Intimate partner violence and its health impact on ethnic minority women	Journal of Women’s Health	191	10 .1089/jwh.2014.4879
7	Arnold T, [16],	Social, structural, behavioral and clinical factors influencing retention in Pre-Exposure Prophylaxis (PrEP) care in Mississippi	Plos One	168	10. 1371/journal.pone.0172354
8	Raviglione M, [17],	Tuberculosis 2015: burden, challenges and strategy for control and elimination	Infectious Disease Reports	166	10. 4081/idr.2016.6570
9	Ataguba Je, [18],	Socioeconomic-related health inequality in South Africa: evidence from General Household Surveys	International Journal for Equity in Health	159	10. 1186/1475-9276-10-48
10	Marshall Bdl, [19],	Formalizing the Role of Agent-Based Modeling in Causal Inference and Epidemiology	American Journal of Epidemiology	149	10. 1093/aje/kwu274

### Trends in publication and citation

**Figure 2.**
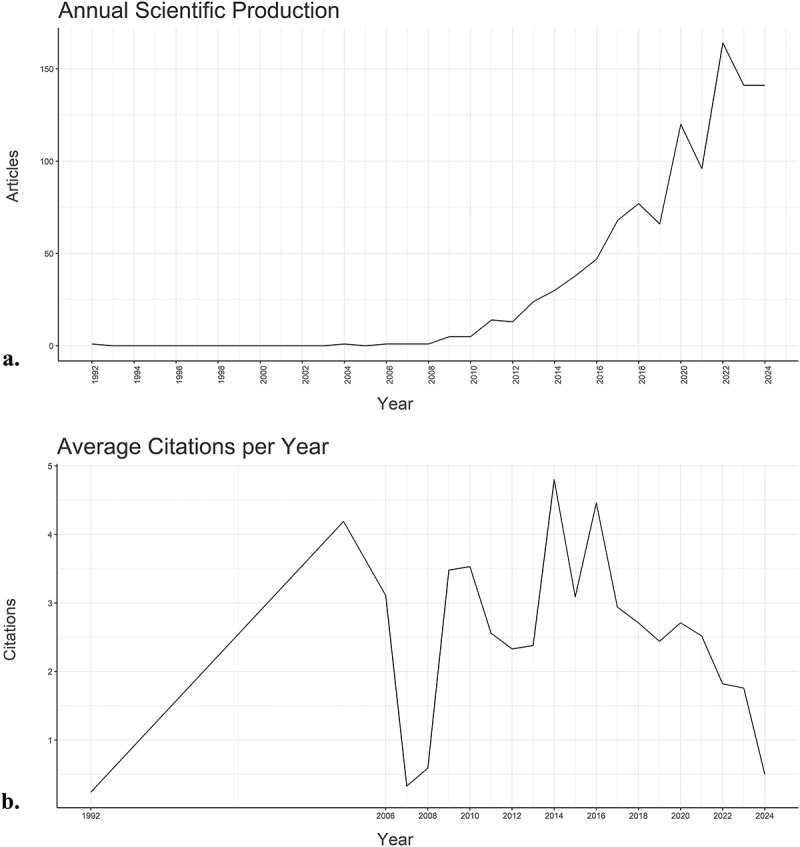
The global annual trends showing (a) the number of published articles and (b) citation counts related to socio-economic burden of HIV infection from 1992 to 2024.

The results demonstrated the increasing volume of publications annually, with 2022 seeing the highest number of published articles (*N* = 164), while 1994–2002 saw no publications at all (*N* = 0). The analysis revealed noticeable fluctuations in the average number of citations per article across different years. For instance, in 2014, there was a notable increase in average citations, peaking at 4.8 per year. In contrast, 1992 experienced the lowest citation average, with 0.2 citations per article recorded. The trends in publications across these years are visually represented in Figure 2(a,b).

**Figure 3.**
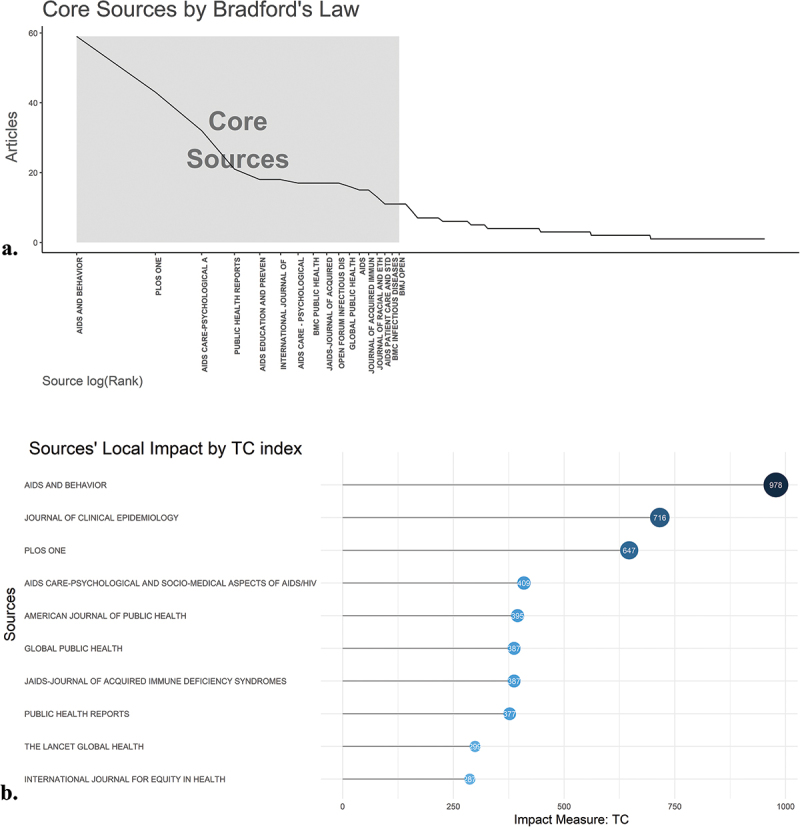
(a) Broadford’s Law was applied to identify three key journals that prominently focus on socio-economic burden of HIV infection from 1992 to 2024. (b) The journals’ local influence is reflected through their total citations during the same period.

Bradford’s Law was employed to analyze the distribution of scientific articles across journals. From this, three core journals were identified as consistently attracting the most research contributions (Figure 3(a)). According to Bradford’s Law, these journals accounted for a substantial portion of the total articles published on socio-economic burden of HIV infection. Among them, ‘Aids and Behavior’ stood out as the most prolific journal, contributing 59 articles, which represents approximately 5.6% of all published articles during the study period. Additionally, local citations from within our dataset revealed that this journal also garnered the most citations, with an impressive total of 978 (Figure 3(b)).

### Leading institutions, authors, countries, and collaboration networks

**Figure 4.**
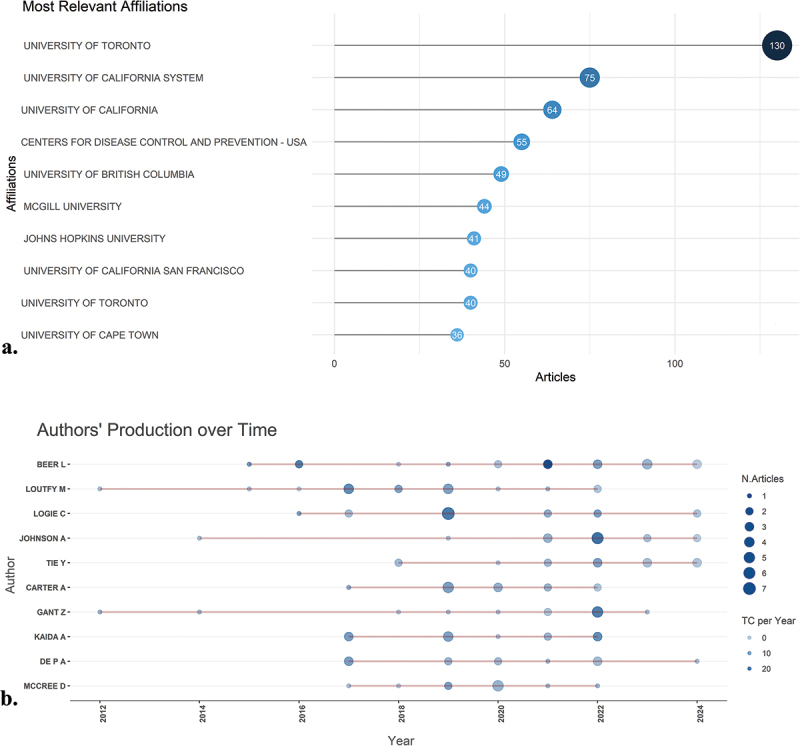
(a) the figure highlights the most active authors, institutions, and countries, along with their networks of collaboration. (b) It showcases the top ten authors, detailing their contributions over time in the field of socio-economic burden of HIV infection from 1992 to 2024.

**Figure 5.**
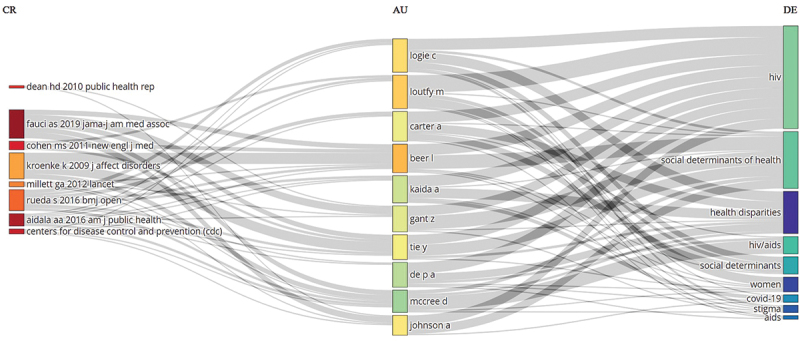
The Three-Fields Plot provides a visualization of the relationships between cited references (CR), authors (AU), and author keywords (DE) in research on socio-economic burden of HIV infection from 1992 to 2024. Abbreviations in the right column (DE) include: HIV, social determinant of health, health disparities, HIV/AIDS, social determinant, women, COVID-19, stigma, and ethics of AIDS.

The University of Toronto in Canada made the most significant institutional contribution to this field, publishing 130 articles, which constitutes 2.5% of the total research output (Figure 4(a)). The authors Beer L. and Loutfy M. were the most prolific, publishing 20 and 17 articles, respectively, accounting for 0.24% and 0.20% of the total number of articles. The authors with the highest number of publications are shown in Figure 4(b). The three-fields plot effectively illustrates the intricate network of relationships among authors, cited references, and author keywords, providing deep insights into the landscape of research on socio-economic burden of HIV infection between 1992 and 2024 (Figure 5).

At the national level, the USA and the Canada were the leading contributors to this field, with 1623 and 650 publications, respectively. United Kingdom (UK), South Africa and Brazil followed with 122, 83 and 78 articles, respectively (Table 2). USA and Canada had 92.2% and 94% of their publications in Single-country publications; hence, they had highest Multiple-country publications number among other countries (*N* = 126 and *N* = 39, respectively). In contrast, Cina, Ethiopia and Spain demonstrated the lowest collaborative research efforts. Moreover, no collaboration was observed among France publications (Figure S1A). Moreover, the USA was also the most cited country by far among other countries with 7673 citations (Figure S1B).

**Table 2.** Leading countries published on socio-economic burden of HIV infection from 1992 to 2024.

Journal	Number of Articles	Multiple Country Publication	Single Country Publication
USA	1623	126	1497
Canada	650	39	611
UK	122	28	94
South Africa	83	13	70
Brazil	78	11	67
Australia	63	16	47
China	31	1	30
Ethiopia	25	1	24
Spain	25	4	21
France	22	0	22

### Keyword analysis, emerging trends, and thematic developments

The analysis utilized Biblioshiny to examine the most frequently occurring author keywords, focusing on terms such as ‘social determinants of health’, ‘human immunodeficiency virus infection’, and ‘HIV infections’. Keywords associated with HIV (‘HIV infections’, ‘human immunodeficiency virus infection’ and ‘acquired immune deficiency syndrome’) and socio-economic burden (‘social determinants of health’ and ‘sexual behavior’) showed upward trends, with 331, 567, 102, 604, and 118 instances in 2024, respectively. The prevalence of terms like ‘social determinants of health’, ‘human immunodeficiency virus infection’ and ‘HIV infections’ remained steady over time, with 604, 567 and 331 instances in 2024. Research on HIV witnessed a significant rise, suggesting growing attention on this disease. The continued interest in the quality of healthcare is evident through keywords related to socio-economic burden of HIV disease (Figure S2A and S2B). A temporal analysis of key terms showed that ‘human immunodeficiency virus infection’ peaked in citation count in 2021, while ‘social determinants of health’ maintained its importance in 2020 (Figure S3).

## Discussion

### Key findings of this study

This study provides a comprehensive examination of the socio-economic burden of HIV, utilizing bibliometric analysis to trace research trends over time. One of the major findings is the significant rise in publications on the socio-economic aspects of HIV infection, especially in the last two decades. This increase parallels the global recognition of HIV as not merely a health issue but also a critical socio-economic concern. Recent research by Dailey et al. (2021) has highlighted similar trends, showing an increase in the number of studies addressing the social determinants of health in relation to HIV [20]. This study confirms that regions such as North America and Europe are leaders in HIV-related research, while regions with a higher burden, such as sub-Saharan Africa, continue to lag behind in research output. This lack of representation is concerning, given that sub-Saharan Africa accounts for more than 60% of the global HIV burden [3,4].

Interestingly, this study also reveals a steady increase in collaboration among researchers from high-income countries, as indicated by the growing number of multi-author publications. This reflects the importance of international cooperation in HIV research, as pointed out by Halid et al. [21], who emphasized that collaborative efforts are crucial for tackling global epidemics. However, the lack of significant collaboration with African nations, despite their high disease burden, underscores a critical gap in the global research agenda.

The findings of this study align with previous bibliometric analyses conducted by Tran et al. [22], which also noted a steady increase in publications focused on the socio-economic implications of HIV. Similar to this study, they observed that high-income countries, particularly the United States, dominate the research landscape. However, what sets this study apart is its focus on the evolution of key research themes, such as the intersection of HIV with social inequality, stigma, and healthcare access. This thematic shift from biomedical research to broader socio-economic factors echoes findings from Figueroa et al. [23], who also reported that recent research is increasingly addressing the socio-political and economic contexts in which HIV spreads.

The identification of key journals such as *The AIDS and Behavior and PLOS ONE* as primary sources for high-impact publications is consistent with bibliometric trends observed by Doan et al. [24] (Table 3). Additionally, the present study highlights the centrality of terms like ‘social determinants of health’ and ‘health disparities’ in recent HIV research, which reflects a growing recognition of the role that socio-economic factors play in shaping health outcomes. John [25] also emphasized the importance of addressing these broader determinants, particularly in the context of marginalized populations disproportionately affected by HIV.

**Table 3.** The top 10 most cited journals on socio-economic burden of HIV infection from 1992 to 2024.

Journal	Total Citation
AIDS and Behavior	978
PLOS ONE	647
AIDS Care-Psychological and Socio-Medical Aspects of AIDS/HIV	409
Public Health Reports	377
Global Public Health	387
JAIDS-Journal of Acquired Immune Deficiency Syndromes	387
BMC Public Health	284
AIDS Education AND Prevention	226
AIDS	166
AIDS Care-Psychological and Socio-Medical Aspects OF AIDS/HIV	92

### Limitations of bibliometric analysis

Although bibliometric analysis provides valuable insights into research trends and collaborations, it has inherent limitations. One of the primary limitations is the reliance on publication and citation counts as proxies for research impact. As noted by Sugimoto et al. [26], highly cited papers are not always indicative of groundbreaking or high-quality research. Furthermore, bibliometric data often lack the depth necessary to fully understand the nuances of the research landscape. For example, this study relies primarily on English-language databases, which may underrepresent research conducted in non-English-speaking countries, particularly those in Africa and Latin America [27].

Additionally, bibliometric analyses tend to focus on quantifiable metrics rather than qualitative assessments of research impact. This can lead to an overemphasis on certain themes or topics that are popular within academic circles but may not reflect the most pressing issues on the ground [28]. To address these limitations, this study complements the bibliometric findings with a detailed review of the literature on HIV pathogenesis and its socio-economic implications.

### HIV/AIDS pathogenesis and socio-economic burden

#### The virus itself

HIV is a retrovirus that targets the immune system, specifically CD4+ T-cells, which are essential for coordinating the body’s immune response. Once inside the host cell, the virus integrates its genetic material into the host’s DNA, leading to the production of new viral particles. Over time, this process depletes the body’s CD4+ T-cells, weakening the immune system and leaving the individual vulnerable to opportunistic infections [29,30]. There are two main types of HIV: HIV-1, which is the most prevalent worldwide, and HIV-2, which is mainly found in West Africa [31].

#### Incubation period

Following initial infection, there is typically a long latency period during which the virus replicates slowly. This asymptomatic phase can last several years, with individuals remaining infectious even though they may not display any symptoms [32,33]. Without treatment, the virus gradually weakens the immune system, leading to AIDS. The incubation period can be influenced by various factors, including the individual’s immune response and whether they receive ART [32].

#### Pathogenesis

HIV pathogenesis is characterized by the progressive destruction of CD4+ T-cells, which are critical for immune function. As the virus replicates, it gradually depletes these cells, impairing the body’s ability to fight infections and diseases. In the absence of treatment, HIV typically progresses to AIDS within 10–15 years [30,34]. AIDS is marked by the development of opportunistic infections such as tuberculosis and certain cancers, which take advantage of the weakened immune system. ART can slow the progression of HIV by reducing viral load, but once the immune system is severely compromised, the risk of life-threatening infections increases [35].

#### Signs and symptoms

The symptoms of HIV can vary depending on the stage of infection. During the acute infection phase, individuals may experience flu-like symptoms such as fever, headache, and swollen lymph nodes. As the disease progresses, these symptoms may worsen, and new complications such as weight loss, chronic diarrhea, and neurological issues may arise [36,37]. AIDS is characterized by severe immune system damage, leading to life-threatening infections and cancer [36].

#### Mortality and life expectancy

The improvements in mortality rates and life expectancy among people living with HIV observed in this study are consistent with global trends reported by UNAIDS (2022) [3]. The availability of ART has been transformative, reducing mortality and improving quality of life for millions. This study echoes findings by Quinn et al. [38], who demonstrated that life expectancy for people living with HIV has increased significantly in countries with widespread ART availability, though it remains below that of the general population. In regions like sub-Saharan Africa, where access to ART is more limited, life expectancy for people living with HIV remains substantially lower than in high-income countries [39]. The persistent gap in life expectancy despite ART availability highlights ongoing disparities in healthcare access, a theme also explored by Bono et al. [40].

#### Socio-economic burden

The socio-economic burden of HIV remains a critical concern, particularly in low- and middle-income countries. The financial costs of managing HIV are immense, both at the individual and societal levels. This study corroborates findings by Smith et al. [41], who showed that HIV-related health-care costs, lost productivity due to illness, and social stigma contribute significantly to economic strain in high-prevalence regions. For individuals, the cost of long-term ART, combined with the need for regular medical monitoring, imposes a heavy financial burden, particularly for those in low-income settings. According to the previous study [42], the economic impact of HIV in sub-Saharan Africa alone has slowed GDP growth in several countries by as much as 2%.

This study further emphasizes the role of stigma in exacerbating the socio-economic challenges of HIV. Stigma not only contributes to social isolation and mental health issues but also impedes access to healthcare and employment. Similar findings were reported by Feyissa et al. [43], who noted that stigma leads to delayed diagnosis and poorer health outcomes, particularly among marginalized groups. Reducing stigma through targeted public health campaigns and legal protections for people living with HIV remains a crucial aspect of mitigating the socio-economic burden of the disease.

The impact of HIV on families and communities is also profound. The death of a household’s primary breadwinner due to AIDS can push families into extreme poverty, creating cycles of economic hardship that can persist for generations [44]. This is particularly evident in countries with high HIV prevalence, where extended family structures often take on the burden of caring for orphans and ill relatives, further straining economic resources. Studies by Gilbert and Walker (2010) have shown that the socio-economic impact of HIV extends far beyond the individual, affecting entire communities and national economies [45].

#### Socio-economic burden: detailed breakdown

- **Health-care Costs**: The financial cost of managing HIV is immense, particularly in resource-limited settings. ART, while life-saving, requires lifelong adherence and regular monitoring, which can be prohibitively expensive for individuals in low-income countries. A 2021 report by the World Health Organization (WHO) estimated that the global cost of HIV care exceeds $20 billion annually. This figure includes the cost of ART, the management of opportunistic infections, and the infrastructure required to deliver care [5].
- **Loss of Productivity**: HIV predominantly affects individuals in their most productive years, leading to significant losses in labor productivity. The economic impact is particularly severe in sub-Saharan Africa, where HIV prevalence is the highest. A study by the International Labour Organization (ILO, 2021) estimated that HIV-related morbidity and mortality have reduced workforce productivity by more than 30% in some high-prevalence countries. This loss of human capital has a direct effect on national economies, slowing GDP growth and exacerbating poverty [46,47].
- **Stigma and Discrimination**: HIV-related stigma continues to be a significant barrier to care and social inclusion. Stigma not only prevents individuals from seeking timely treatment but also affects their employment opportunities and social relationships. Studies by Logie and Turan (2020) have shown that stigma is particularly harmful in regions where HIV prevalence is high but public awareness remains low. Legal protections for people living with HIV, along with public education campaigns, are critical to reducing the stigma and improving health outcomes [48].
- **Impact on Families**: The socio-economic burden of HIV extends beyond individuals to entire families and communities. In many cases, the death of a primary income earner due to AIDS forces families into poverty. This economic strain is compounded when children are orphaned and must rely on extended family networks or the state for support. A study by Evans and Miguel (2021) found that in high-prevalence regions, such as rural South Africa, households affected by HIV/AIDS experience significantly higher levels of poverty and food insecurity [49].

#### Solutions to overcome the socio-economic burden

To alleviate the socio-economic burden of HIV, several interventions are necessary:

- **Scaling Up ART Programs**: Expanding access to ART is essential to reducing HIV-related mortality and improving quality of life. The WHO (2024) has called for increased funding and infrastructure development to ensure that ART is accessible to all who need it, particularly in low-income countries [5].
- **Reducing Stigma**: Public health campaigns aimed at reducing stigma have proven effective in improving health outcomes for people living with HIV. Countries like South Africa have launched successful anti-stigma initiatives that have reduced discrimination and encouraged more people to seek testing and treatment [50].
- **Addressing Social Determinants of Health**: Policies that address the root causes of HIV, such as poverty, inequality, and lack of education, are critical to reducing the socio-economic impact of the disease. Research by Jeffries and Henny (2019) has shown that interventions targeting social determinants can significantly reduce HIV transmission and improve health outcomes in high-prevalence areas [51,52].
- **Strengthening International Collaboration**: Global partnerships are crucial for combating HIV, particularly in low-income countries where resources are limited. International collaborations can provide the funding, expertise, and political support necessary to implement effective HIV prevention and treatment programs [53].

### Future prospects

The future of HIV research and intervention lies in a multidisciplinary approach that addresses both the biomedical and socio-economic aspects of the disease. Advances in ART, such as long-acting injectable therapies, offer new hope for improving adherence and reducing the burden of daily medication [54]. Additionally, ongoing research into HIV vaccines remains a key priority, with several candidates currently in clinical trials [3,4].

Furthermore, it is critical to address the research gap in low- and middle-income countries. Strengthening research capacity in regions like sub-Saharan Africa will ensure that local challenges are addressed and that the global research agenda reflects the needs of all affected populations. Expanding international research collaborations, as well as providing targeted funding for capacity building, will be essential for achieving these goals [55].

Future bibliometric studies must prioritize linguistic inclusivity to capture the full spectrum of global research contributions. Expanding database search criteria to include non-English publications will significantly enhance the scope and representation of studies from regions like Latin America, Asia, and non-Anglophone Africa [56]. Leveraging multilingual databases, such as SciELO, Russian Science Citation Index, and CNKI, alongside Scopus and Web of Science, will ensure a more balanced dataset. Moreover, employing machine translation tools can facilitate the inclusion of articles in languages other than English. International collaborations with researchers proficient in local languages can also aid in contextualizing findings, thereby enriching global HIV research. Such approaches will ensure that insights from diverse linguistic and cultural contexts are integrated, leading to more equitable research outcomes.

## Conclusion

This bibliometric analysis of HIV socio-economic burden research from 1992 to 2024 reveals significant progress in understanding the complex interplay between HIV and socio-economic factors. The study highlights the growing recognition of social determinants of health in HIV research, as well as increased international collaboration, particularly among high-income countries. However, it also exposes critical gaps, notably the underrepresentation of research from high-burden regions like sub-Saharan Africa. The findings underscore the need for more inclusive global research collaborations and a sustained focus on addressing socio-economic disparities in HIV prevention and treatment. As the field evolves, future research should prioritize integrating perspectives from diverse geographical and socio-economic contexts to develop more effective, equitable strategies for managing the global HIV epidemic. This comprehensive analysis serves as a valuable resource for researchers, policymakers, and health-care professionals working to mitigate the socio-economic impact of HIV worldwide.

## Supplementary Material

Supplementary file_.docx

## Abbreviations

HIV: Human Immunodeficiency Virus
AIDS: Acquired Immunodeficiency Syndrome
ART: Antiretroviral Therapy
MeSH: Medical Subject Headings
WOS-CC: Web of Science Core Collection
USA: United States of America
UK: United Kingdom

## Generative artificial intelligence (AI) statement

This article was written entirely by the authors. ChatGPT-4 was utilized exclusively for language editing purposes, and no other parts of the content were generated or influenced by any AI software.
