# Supplementary material for: Exploring socio-economic dimensions in HIV research: a comprehensive bibliometric analysis (1992–2024)
Source: Glob Health Action. 2025 Mar 12;18(1):2474787. doi: 10.1080/16549716.2025.2474787 (PMC11905308; doi:10.1080/16549716.2025.2474787)
Supplement: Supplementary file_.docx [file ZGHA_A_2474787_SM9167.docx]

**Exploring Socio-Economic Dimensions in HIV Research: A Comprehensive Bibliometric Analysis (1992–2024)**

Running title: Socio-Economic Factors in HIV

**Lyudmila Yermukhanova^a^, Marat Kuzembayev^a,^*, Akkumis Salkhanova^b^, Nazerke Narymbayeva^c^, Aigul Tazhiyeva^d^, Dinara Nurgalievna Makhanbetkulova^e^, Alireza Afshar^a,^***

^a^West-Kazakhstan Marat Ospanov Medical University, Aktobe, Kazakhstan. [yermukhanova@zkmu.kz](mailto:yermukhanova@zkmu.kz) (L.Y.); [marat.kuzembaev@gmail.com](mailto:marat.kuzembaev@gmail.com) (M.K.); [Alireza.af2017@gmail.com](mailto:Alireza.af2017@gmail.com) (A.A.);

^b^Kazakh Academy of Nutrition, Almaty, Kazakhstan. [asalkhanova@zdrav.kz](mailto:asalkhanova@zdrav.kz) (A.S.);

^c^Kazakhstan Medical University "KSPH", Almaty, Kazakhstan. [n.narymbay@gmail.com](mailto:n.narymbay@gmail.com) (N.N.);

^d^Kazakh National Medical University named after S.D. Asfendiyarov, Almaty, Kazakhstan. [tazhiyeva@mail.ru](mailto:tazhiyeva@mail.ru) (A.T.);

^e^Head of the department Nursing, Kazakh National medical university, Almaty, Kazakhstan. [makhanbetkulova.d@kaznmu.kz](mailto:makhanbetkulova.d@kaznmu.kz) (D.N.M);

* **Correspondences:**

Alireza Afshar, West-Kazakhstan Marat Ospanov Medical University, Aktobe, Kazakhstan; Email: [Alireza.af2017@gmail.com](mailto:Alireza.af2017@gmail.com)

Marat Kuzembayev, West-Kazakhstan Marat Ospanov Medical University, Aktobe, Kazakhstan.; Email: [marat.kuzembaev@gmail.com](mailto:marat.kuzembaev@gmail.com)

# **Supplementary Tables**

## **Table S1.** Queries for search in Web of Science (WOS) and Scopus databases. #1 This is the keywords related to Social Determinants of Health and its synonyms extracted from MeSh database. #2 This one is HIV and its synonyms extracted from MeSH, and #3 is a brief code for merging queries in WOS database.

| Query number | Keywords for search |
| --- | --- |
| #1 | “Social Determinants of Health”OR”Socio-Economic Determinants of Health”OR”Socio Economic Determinants of Health”OR”Structural Determinants of Health”OR”Commercial Determinants of Health” |
| #2 | “HIV”OR”HTLV-III”OR”Human Immunodeficiency Virus”OR”Immunodeficiency Virus, Human”OR”Immunodeficiency Viruses, Human”OR”Virus, Human Immunodeficiency”OR”Viruses, Human Immunodeficiency”OR”Human Immunodeficiency Viruses”OR”Human T Cell Lymphotropic Virus Type III”OR”Human T-Cell Lymphotropic Virus Type III”OR”Human T-Cell Leukemia Virus Type III”OR”Human T Cell Leukemia Virus Type III”OR”LAV-HTLV-III”OR”Lymphadenopathy-Associated Virus”OR”Lymphadenopathy Associated Virus”OR”Lymphadenopathy-Associated Viruses”OR”Viruses, Lymphadenopathy-Associated”OR”Virus, Lymphadenopathy-Associated”OR”Human T Lymphotropic Virus Type III”OR”Human T-Lymphotropic Virus Type III”OR”AIDS Virus”OR”AIDS Viruses”OR”Virus, AIDS”OR”Viruses, AIDS”OR”Acquired Immune Deficiency Syndrome Virus”OR”Acquired Immunodeficiency Syndrome Virus” |
| #3 | #1 AND #2 |

## **Table S2.** Codes were used to merge Scopus and Web of Science exported data in RStudio. This code was used to merge the data extracted from Scopus and WOS, which were initially separate files.

| Command code |
| --- |
| library(bibliometrix)  library(openxlsx)  ## importing web of science dataset  web_data<-convert2df("abs.txt")  ## importing scopus dataset  scopus_data<-convert2df("abs.bib",dbsource="scopus",format="bibtex")  ##combined both datasets  combined<-mergeDbSources(web_data,scopus_data,remove.duplicated=T)  ##exporting file  write.xlsx(combined,"combinedabs.xlsx") |

# **Supplementary Figures**


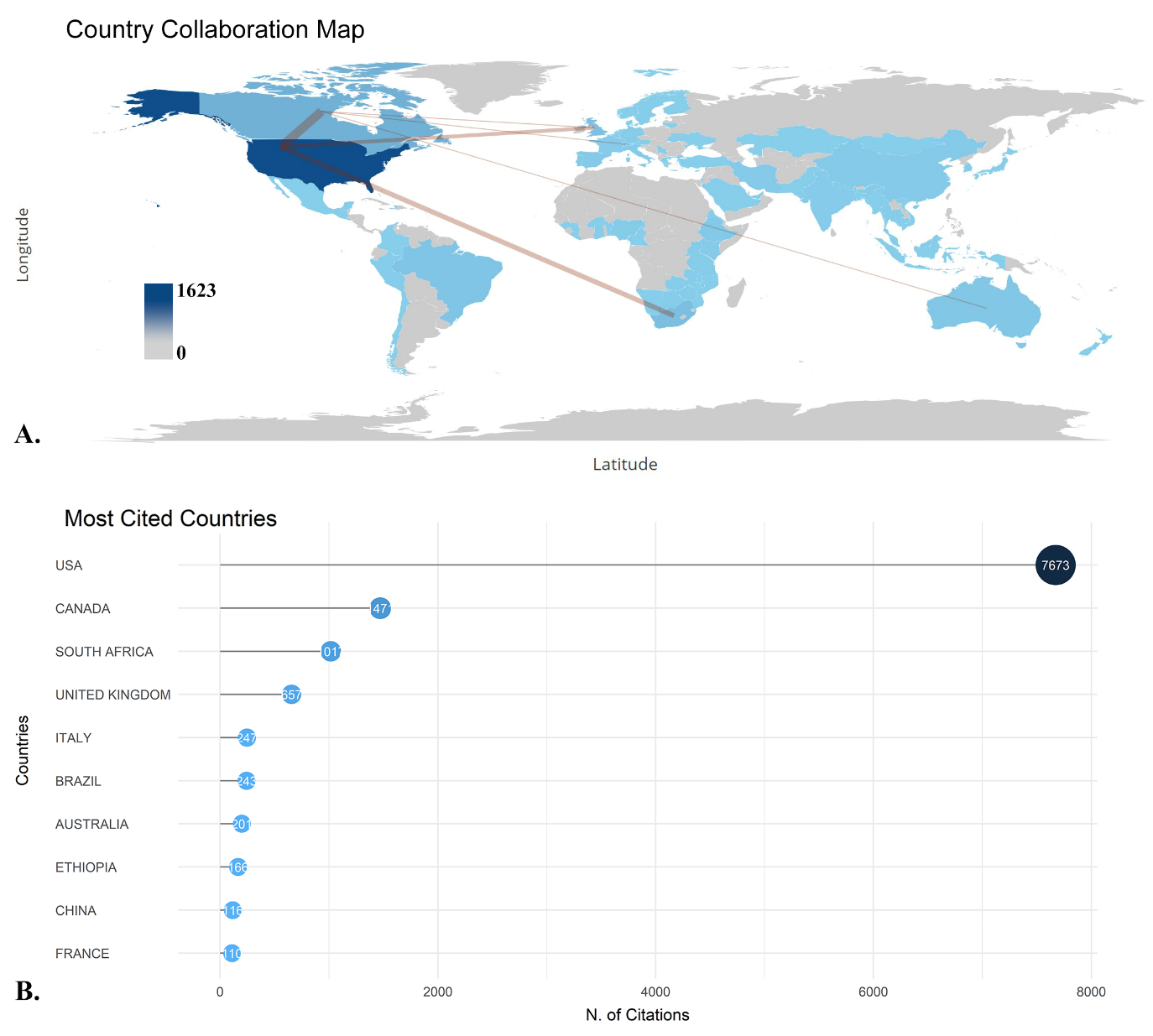


## **Figure S1.** (A) A global collaboration map on socio-economic burden of HIV infection from 1992 to 2024. Darker shades indicate a higher number of publications per country, while the arrows represent the strength and extent of international collaboration. (B) Number of citations of each country of most cited countries.


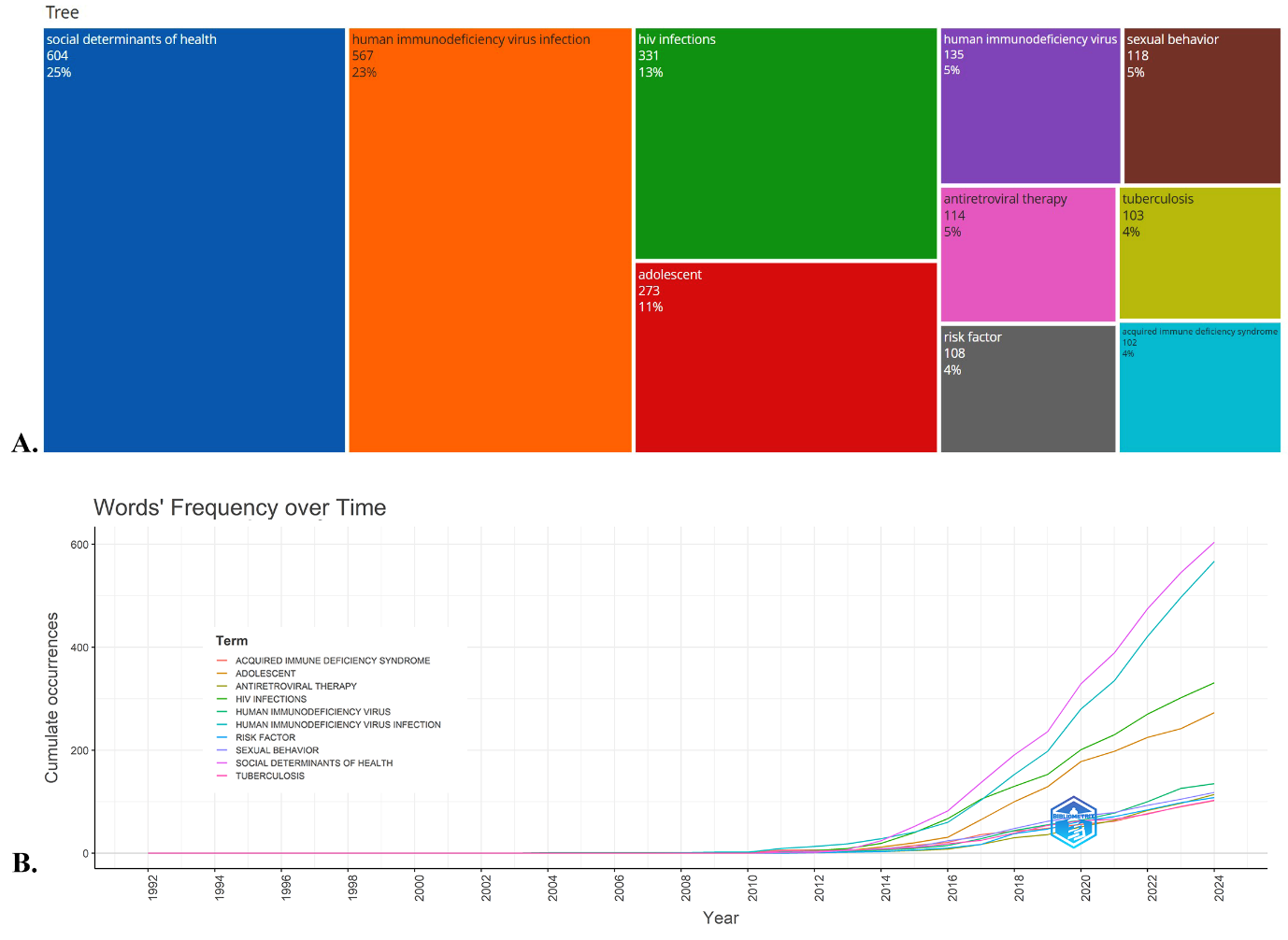


## **Figure S2.** (A) The TreeMap and (B) the scatter plot display the ten most commonly used keywords in research concerning socio-economic burden of HIV infection from 1992 and 2024. (A) The Treemap shows each most frequent keyword with a specific color and its percentage of frequency.


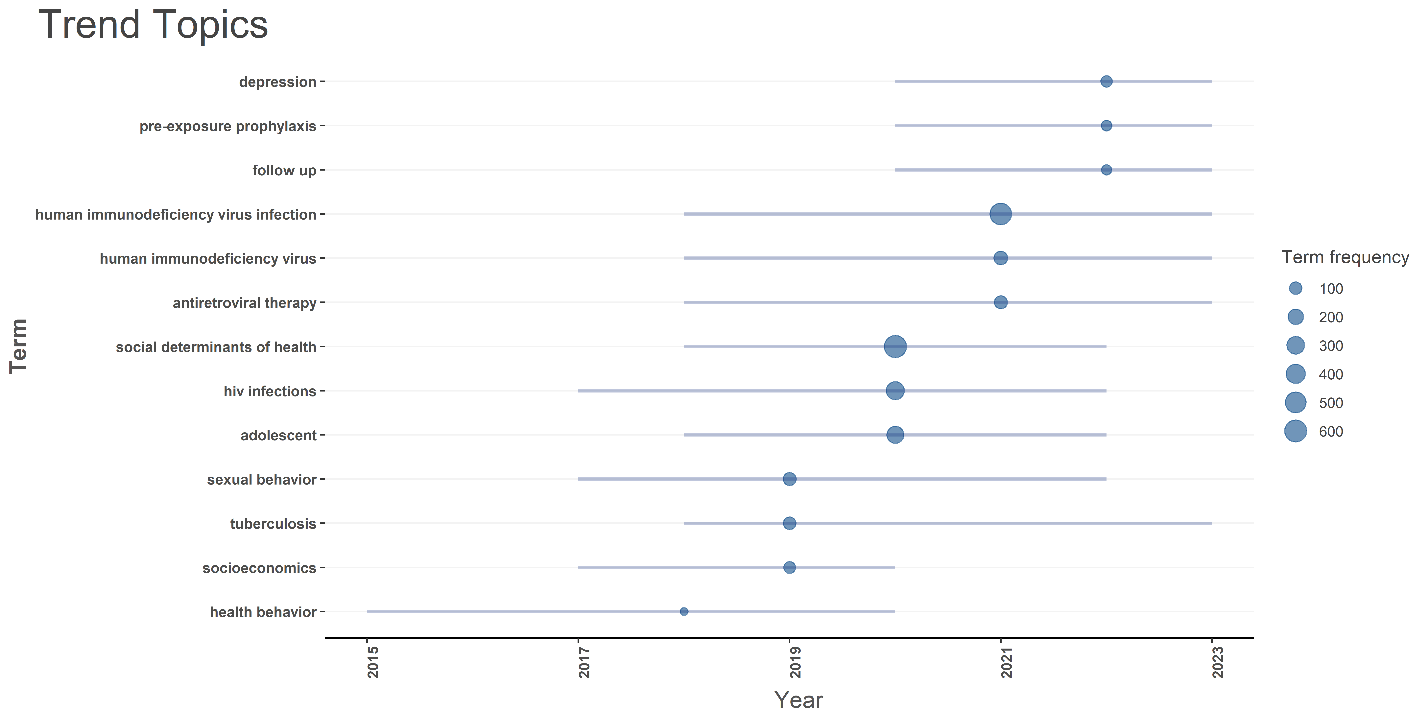


## **Figure S3.** The timeline of emerging research topics is shown, with each sphere representing the peak frequency of a specific topic, and the lines tracing the years during which the topics were most prominent.
